# Use of prior knowledge for the analysis of high-throughput transcriptomics and metabolomics data

**DOI:** 10.1186/1752-0509-8-S2-S2

**Published:** 2014-03-13

**Authors:** Polina Reshetova, Age K Smilde, Antoine HC van Kampen, Johan A Westerhuis

**Affiliations:** 1 Biosystems Data Analysis, Swammerdam Institute for Life Sciences, University of Amsterdam, Science Park 904, 1098 XH, Amsterdam, the Netherlands; 2 Bioinformatics Laboratory, Academic Medical Center, University of Amsterdam, P.O. Box 22700, 1100 DE, Amsterdam, the Netherlands; 3 Netherlands Bioinformatics Centre, Geert Grooteplein 28, 6525 GA, Nijmegen, the Netherlands

## Abstract

**Background:**

High-throughput omics technologies have enabled the measurement of many genes or metabolites simultaneously. The resulting high dimensional experimental data poses significant challenges to transcriptomics and metabolomics data analysis methods, which may lead to spurious instead of biologically relevant results. One strategy to improve the results is the incorporation of prior biological knowledge in the analysis. This strategy is used to reduce the solution space and/or to focus the analysis on biological meaningful regions. In this article, we review a selection of these methods used in transcriptomics and metabolomics. We combine the reviewed methods in three groups based on the underlying mathematical model: exploratory methods, supervised methods and estimation of the covariance matrix. We discuss which prior knowledge has been used, how it is incorporated and how it modifies the mathematical properties of the underlying methods.

## Background

High-throughput technologies such as DNA microarrays in transcriptomics and mass spectrometry in metabolomics produce large amounts of experimental data, where each sample is characterized by the expression levels of thousands of genes, or concentration levels of hundreds to thousands of metabolites respectively. This high number of variables gives a unique chance to catch a broad range of biological processes but, at the same time, poses significant challenges to statistical methods of analysis. First of all, traditional statistical methods highlight relationships among variables based only on mathematical criteria (e.g. maximizing variance or correlation among variables) and thereby do not always distinguish between correlations from biological origin and chance correlations that may arise because of the high dimensionality of the data and measurement noise. Secondly, biological differences of the subjects in the study produce variations in gene expression values and metabolite concentrations in experiments. Very often such biological variation is not of primary interest and not under control of the researcher. Therefore, a challenge of statistical methods in transcriptomics and metabolomics is to distinguish between the different variation sources.

Recently, new methods have appeared that use prior knowledge of the biological system to guide the statistical analysis to enhance discovery of new biology while reducing the detection of spurious relationships. In addition, prior knowledge may be used to check consistency of the available knowledge and experimental data to fill in possible gaps or add more detail. We focus our review on approaches that incorporate prior knowledge about the relationship between the genes or between metabolites to achieve an optimal balance between mathematical criteria and known biology. The relationships among variables (genes or metabolites) can be determined, for example, from public databases that contain results of previous experimental data analysis. For example, the KEGG [1] database contains information about metabolic pathways, GO [2] contains annotation of gene products, the TRANSFAC [3] database contains information about transcription factors, their binding sites, and target genes. We are specifically interested in how each method manages the balance between the discovery of new biology and, using prior knowledge, forcing the results towards existing biology.

In this review, we focus on high dimensional supervised and unsupervised data analysis methods that include prior knowledge into the mathematical model used for the analysis of metabolomics or transcriptomics data. Methods for genomics and proteomics also have been developed (see for example [4,5]) but they are out of the scope of our review. To the best of our knowledge, our review is the first that provides a comprehensive overview of strategies using prior biological knowledge in metabolomics and transcriptomics data analysis. In the remainder of this text we will refer to metabolomics and transcriptomics data as 'omics' data.

To structure this review, we classified the reviewed methods into three groups, based on the mathematical approach and whether the method is unsupervised or supervised. We distinguish three groups of mathematical approaches. The first group comprises component models that reduce the dimensionality of the data by constructing latent variables from the observed genes or metabolites. The second group comprises cluster models that use similarity measures to group related genes or metabolites, and the third group comprises covariance methods that primarily aim to estimate variances/correlations among the genes or metabolites. We wittingly have not grouped the methods based on the used type of prior knowledge. As can be seen from our review, the same type of prior knowledge is utilized by a range of mathematical methods. In section Exploratory methods we discuss unsupervised methods (component based models and clustering methods). These methods explore data and describe the major drivers underlying the observed data structure. In section Supervised classification methods we discuss supervised methods for finding a classification function that predicts class labels. In section Covariance matrices we discuss methods that estimate the covariance matrix. For each section we provide additional figures [see Additional file 1].

### Two phases of the analysis of high dimensional data

The term model has many interpretations in the bioinformatics literature. In the context of this review we define a 'model' as a statistical or mathematical representation of omics data. Each model has specific estimated parameters, for example, principal components in component models or coefficients in a regression model. Omics data is written as a two-way matrix **X **with *I *rows representing genes or metabolites, and *J *columns representing samples (e.g. subjects, tissues, treatments, diseases). *J *is usually much smaller than *I*. In this review we will focus on data analysis methods that handle two-way data. To facilitate the discussion and comparison of data analysis methods that use prior knowledge, we consider two phases in the analysis of omics data:

1. Definition of a model and estimation of the model parameters.

2. Interpretation of the model parameters in terms of biological knowledge.

Prior biological knowledge can be incorporated in each of these two phases. In the second phase the prior information is used to facilitate or even enable interpretation of the data analysis result. Examples of such methods are gene set enrichment analysis [6] and metabolite set enrichment analysis methods [7]. The enrichment methods have been extensively reviewed by others [8,9]. In this paper we focus on the first phase in which the model parameters are estimated.

**Figure 1:**
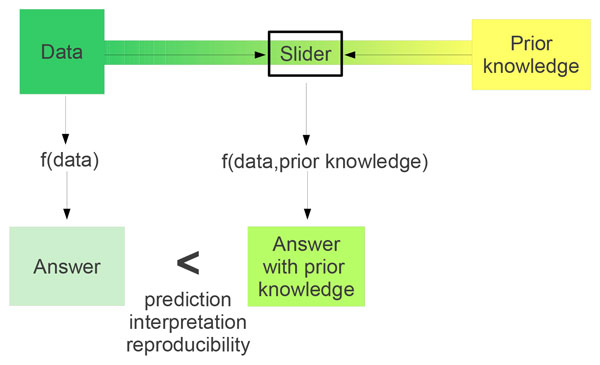
**A general scheme of data analysis methods**. *f(data) *is a function that does not include prior knowledge. *f(data, prior knowledge) *is a function that includes prior knowledge. "Answer with prior knowledge" gives a better predictive model, is easier interpretable and/or more reproducible than "answer" without prior knowledge. The slider controls the strength of the influence of prior knowledge on the result.

The inclusion of prior knowledge in data analysis implies that we have to weight the importance of the data against the importance of the biological knowledge. This is visualized by the slider in Figure 1. The methods discussed in this review implicitly or explicitly (using a weight factor) deal with this balance. Inclusion of prior knowledge aims to emphasize the known relationships between the genes or metabolites while eliminating spurious variation among these variables. However, it may also limit the possibility to make new discoveries if we put too much emphasis on the already known biology. The main challenge is to find an optimal position for the slider such that new discoveries can be made from the data that are in agreement with current biological knowledge. The methods reviewed in this paper follow different strategies to set this balance.

### Exploratory methods

#### Component models

Component models are used in omics data analysis to extract and represent the most informative changes in the experimental data among different conditions or samples. Recently, new approaches have appeared that use prior information such as regulation networks, protein networks, and metabolic networks to highlight the most valuable changes that are related to the question of the study. The basic equation of component models is

(1) $$X=AF^{T}+E$$

where **X **is an *I *by *J *data matrix that consists of *I *variables (genes or metabolites) and *J *samples. Matrix **A **contains the linear components (summarizers) of the data **X**. Matrix **F **contains the weights of these components in each sample. The residual matrix **E **contains the part of the data not explained by the model. Matrices **A **and **F***^T ^*are estimated to maximize the fraction of data variation that is explained by the components. The combination of columns in **A **and **F***^T ^*are called principal components and are required to be orthogonal (Principal Component Analysis) or independent (Independent Component Analysis). In PCA matrices **A **and **F***^T ^*are estimated in a least squares sense to minimize the sum of squares of the residuals (Equation 2).

(2) $$\underset{A,F}{\min}{\left[∥X-AF^{T}∥\right]}^{2}$$

The prior information is translated into the mathematical model by applying various restrictions on the elements of **A **and **F***^T^*. The restrictions are a predefined range of certain elements in **A **and **F***^T ^*or dependencies of some elements on other elements in **A **or **F***^T^*. Because of the restrictions, the new principal components are no longer forced to be orthogonal and may deviate from their standard requirements to reflect better the underlying biological processes. We define two concepts of incorporation of prior knowledge in a component model.

The first concept is based on a relatively simple idea. Metabolites or genes are split into two groups with the ones on which the focus will be in one group (X2) and the remainder in another (X1). The analysis also shows metabolites or genes from the first group, which follow profile patterns of the second group. Van den Berg *et al*. [10] adjusted consensus PCA to implement the concept. Equation 3 and Figure 2 (Additional file 1) demonstrate the method.

(3) $$\left[\begin{array}{c} w_{1}\cdot X_{1}\\ w_{1}\cdot X_{1} \end{array}\right]=\left[\begin{array}{c} A_{1}\\ A_{2} \end{array}\right]F^{T}+\left[\begin{array}{c} E_{1}\\ E_{2} \end{array}\right]$$

where **X***_1 _*and **X***_2 _*are two parts of **X**. **X***_2 _*contains a small group of metabolites that are thought to be important for the problem under the study. Initially, weights *w*_1 _and *w*_2 _were used to compensate the small size of matrix **X***_2_*. This weights were set to the square root of the sum of squares of the corresponding matrix. Consequently, the total variation in each matrix became "1" and equally important for explaining variation in the data. We suggest the weights may also be used as the slider (Figure 1) to put more emphasis on known relationships among metabolites in **X**_2_. The method was applied on experimental data from a phenylalanine overproducing strain and wild type strain of *E. coli *that contained measurements of metabolites under 28 conditions. The authors selected the phenylalanine biosynthesis pathway for the subset of genes in the matrix **X**_2 _and showed that the method was able to successfully identify large common effects between the metabolome in the matrix **X***_1 _*and the specific metabolites in the matrix **X***_2_*. The second concept to include prior knowledge predefines variations, which must be described. For example, Network Component Analysis (NCA) of Liao and colleagues is forced to catch variation within a gene regulatory network [11]. For that the method specifically searches for changes in expression level of genes that are known to be regulated by a transcription factor. Variation caught by the method is interpreted as the activity of the transcription factor in the network during the experiment. Therefore, the method uses known qualitative information about regulatory networks topology to generate quantitative network information on the connection strength between genes and transcription factors while decomposing experimental data. The formula of the method is

(4) $$X=A_{TF}\cdot F^{T}+E$$

where each column in **A***_TF _*is forced to represent the effect on genes of a single transcription factor by putting zeros representing that the specific gene is not regulated by that specific transcription factor (Figure 3 in Additional file 1). The further estimation of the matrix **A***_TF _*is made only for elements that are not restricted to be 0. Thus only the genes that are regulated by the corresponding transcription factor will have parameters in **A***_TF _*estimated and the values in the matrix **F***_T _*are considered as the activity of that transcription factor in each sample.

The authors analyzed experimental data of a cell cycle regulation in *S. cerevisiae *and focused the analysis on 11 transcription factors that are known as regulators in the cell-cycle. NCA successfully revealed the role of each transcription factor; in contrast, the gene expression ratios of the transcription factors do not suggest their important role.

Later it was shown that NCA suffered from many false connections between genes and transcription factors in the prior knowledge. Therefore a realization of the slider that would control inputs of biological and mathematical constraints was needed. Yu and Li proposed to use the high-confident part of the prior knowledge to build a model [12]. The model is finalized later through iterations between an estimation of components on experimental data and an estimation on the low-confident part of the prior knowledge. The authors argued that the iteration process allowed to reduce the influence of false connections on the model. As proof the authors report two regulatory networks under different growth conditions for *S. cerevisiae*. For the same purpose, Tran *et al*. suggested to combine stepwise regression and NCA in an iterative approach [13]. The authors argue that their algorithm overcomes the problems of NCA in the analysis of large networks where multiple transcription factors regulate a single gene. Moreover, the authors argued that NCA could not be used in the case when the number of experiments was very small and their method overcame this limitation. The method was demonstrated on a network that contains 70 transcription factors, 778 genes, and 1423 edges between the transcription factors and genes.

Grey Component Analysis (GCA) is the first implementation of the slider that actually allows to choose how much trust is given to the prior knowledge [14]. The topology of gene regulatory networks is used in the same way as in NCA. But how strict the analysis has to follow the prior knowledge is defined by a soft penalty approach. The penalty approach allows using the GCA method for two purposes. If the penalty is strict the decomposition is biased towards prior knowledge. If the penalty is soft the method analyzes the consistency of the data and the prior knowledge. By varying *λ *it is possible to show how well the data follows the prior knowledge and where it does not follow it anymore.

To implement the idea, GCA minimizes the combined sum of squares of the model residual and the penalty

(5) $$\underset{A,F}{\min}\left[\left||X-AF^{T}|{|}^{2}+\lambda ||W\circ {\left(A-A^{true}\right)}^{2}|\right|\right];\lambda \geq 0$$

where matrix **A **is defined according to the given prior knowledge, but the zeros applied to the matrix **A **in NCA method are allowed to be small values in GCA. The authors argued that in noisy data such as omics data, enforcing real zeros might lead to the mis-estimation of the nonzero values. The added part $\lambda ||W\circ {\left(A-A^{true}\right)}^{2}||$ is the penalty. Matrix **A***^true ^*is the structure as applied in NCA, **A **is the estimated matrix and **W **is an indicator matrix which assures that the penalty is only active on the positions in **A **where **A***^true ^*has zeros. The parameter *λ *determines how much emphasis the method puts to fit the data and how much to follow the prior knowledge in **A***^true^*.

Above we discussed various component models. Table 1 in Additional file 2 summarizes our overview.

#### Cluster models

Cluster analysis aims to construct groups of genes or metabolites that share a biological factor such as a common function or co-regulation by transcription factors. Traditional cluster algorithms base their similarity score only on measured data (gene expression values or metabolite concentrations) and discard known relationships between genes or metabolites. This may, for example, result in clusters of genes/metabolites that exhibit similar profiles across samples but are not necessarily co-regulated, do not have similar functions, or do not participate in the same pathway. Here we describe three concepts of including prior knowledge into clustering:

1. Adjusting the distance measure by including prior knowledge.

2. Improving K-means clustering for variables with similar profiles within one regulatory pathway.

3. Extending model-based clustering by increasing the probability of grouping variables with similar prior knowledge.

These concepts are discussed in more detail below.

The first concept adjusts the clustering distance measure between variables. A distance between variables based on prior knowledge is calculated and added to the data based distance. Based on the combined score the hierarchical tree will cluster variables with both similar experimental profiles and prior knowledge. Figure 4 in Additional file 1 shows the first concept where similarity between GO annotation is used as the prior knowledge distance measure. The slide ruler naturally fits the concept.

The first implementation of the concept was done in a publication of Cheng *et al*. [15]. To achieve the goal the method uses similarity in the GO annotation between genes. GO has a hierarchical structure where more general functional terms are located closer to the root, while more specific terms are located closer to leafs. The authors assumed that the first common ancestor of two terms that is closer to leafs reflects a larger functional similarity of the corresponding genes. The formula of the method is

(6) $$d_{ii^{\prime }}=s_{ii^{\prime }}+g_{ii^{\prime }}$$

where *s_ii' _*is the gene expression similarity score between gene *i *and *i'*, calculated as Euclidian distance between the expression profiles of gene *i *and gene *i'*, and *g_ii_' *is the annotation similarity between GO terms of two genes that is based on the GO terms common ancestor. The authors showed that a strong correlation between biological functions and expression profiles led to a cluster. Genes that had close expression patterns but did not have similar annotation were separated.

R. Kustra and A. Zagdanski improved this approach by including a weight factor that balances the contribution of profile distances and the prior knowledge [16]. The overall distance between two genes i and i' was defined as

(7) $$d_{ii^{\prime }}=\lambda s_{ii^{\prime }}+\left(1-\lambda \right)g_{ii^{\prime }};0\leq \lambda \leq 1$$

where the gene expression similarity *s_ii' _*is given by the Pearson's correlation between gene profiles; *g_ii' _*is the GO annotation similarity; *λ *represents the slider. This method also utilizes the hierarchical structure of the GO tree, but in contrast to the method of Cheng who used the common ancestor, this method uses the Information Content of each node. The authors suggested that the GO similarity measure would diminish spurious perturbations in gene expression levels and would lead to more meaningful clusters by focusing the analysis on the known biology. To study the influence of prior knowledge the authors clustered 3224 yeast genes from 424 microarray experiments. Specifically, the authors proposed to use a protein-protein interaction based measure to asses the biological relevance of clusters for *λ *= 0.0, 0.25, 0.5, 0.75, 1.0. However, since the protein-protein interactions also reflect functional relationships between the genes, it can not be used as an unbiased measure to evaluate the incorporation of GO annotations as prior knowledge. As expected the protein-interaction score increased for smaller *λ*, i.e., stronger influence of the GO annotations. Consequently, it was not possible to suggest a good value of *λ*. An additional stage of validation conducted by a biologist or a new measure of biological relevance of clusters were required. Whereas the cluster methods we have discussed so far use GO information, Hanish *et al*. incorporated metrics on metabolic and regulatory pathways from KEGG into the distance function [17]. The distance function assigns small values to pairs of genes, which are close in a network and show similar expression patterns. Genes which are far apart in the network and are not co-regulated or even oppositely regulated are assigned large values. The distance function emphasizes genes that are co-regulated within pathways.

The proposed model for the distance between two genes is

(8) $$d_{ii^{\prime }}=1-0.5*\left(g_{ii^{\prime }}+s_{ii^{\prime }}\right)$$

where *s_ii' _*is a Pearson correlation based measure and *g_ii' _*is a measure based on the 'minimal degree' of a path between two genes in a metabolic pathway. Both measurements were adapted in order to combine the Pearson correlation and the minimal degree to one joint function that would emphasize genes with a high expression profile correlation and which are tightly linked within a pathway. Compared to a distance measure based on either the correlation or minimal degree, this compound distance compensates for biased results due to, for example, very high profile correlations or missing pieces of prior knowledge. The degree of path is calculated as the sum of incident edges of all nodes between two genes. Note that the authors took a minimal path without hubs, because the hubs are considered to be unspecific or ubiquitous molecules and thus unimportant or misleading for the method. The method does not implement the slide ruler with an explicit weight factor but gives an equal importance for both expression data and prior knowledge.

In hierarchical clustering, the final clusters are defined by horizontally cutting the branches of the tree at a certain level. This may also be a non-trivial process. Dotan-Cohen and co-authors proposed a tree snipping algorithm that construct clusters by cutting selected edges at different levels [18]. This method uses GO terms to annotate each node and provides a novel partitioning of the cluster tree in order to have genes with similar GO annotation in one or closely related clusters. More specifically, during the procedure a GO label list of each leaf of a subtree is compared to the annotation of the corresponding cluster. A leaf with the most dissimilar list of labels will be excluded from the subtree while nodes from close subtrees and similar labels will be included. In the first step, the method builds the hierarchical tree without using the prior knowledge. Subsequently, the method changes the original grouping by incorporating the prior knowledge into the partitioning function. We note that the authors assumed any types of labels and not specifically GO annotation. For example, the transcription factors known to regulate genes can be used. For that reason the tree snipping algorithm does not utilize the hierarchical graph information that is specific for GO. Considering an improvement of other methods by including the graph information (as in [15]) we expect that it might give a better result for the tree partitioning algorithm as well. We also note that the method can be directly used in the field of metabolomics where the partitioning may be improved by metabolic networks, or biological annotation.

The second concept of incorporation of prior knowledge in clustering does not explicitly use prior knowledge as a similarity measure. Instead, in a first step genes are grouped according to prior knowledge and, subsequently, similarity among gene expression profiles within a single group is used to improve the clustering. Following this concept, Tseng *et al*. proposed a clustering method PW-Kmeans (Penalized and Weighted K-means) that extends the K-means method by incorporating GO functional annotations [19] (Figure 5 in Additional file 1). The method groups genes according to known functional annotation from GO and then assigns a weight to each gene. The weight reflects how well the gene expression profile conforms to expression profiles of all others genes in its *a priori *defined group. High expression profile similarity among genes with common functional annotation results in small values of their weights and consequently in tight clusters. In addition, the method introduces a noise cluster that contains all scattered genes, which do not follow expression profiles of other genes with similar GO annotation.

The method adapts the loss function in the following way

(9) $$W\left(C;k;\lambda \right)=\overset{K}{\underset{k=1}{\sum }}\underset{x_{i}\in C_{k}}{\sum }w\left(x_{i};L\right)d\left(x_{i},C_{k}\right)+\lambda \text{|}S\text{|}$$

where *K *is the number of clusters; *d*(*x_i_*, *C_k_*) is the distance between gene *i *expression profile *x_i _*and the mean of the cluster *C_k_*; *w(x_i_*; *L) *is the weight that codes the prior knowledge; *λ *is a penalty term that forces scattered variables in a separate cluster *Cs*; *|S| *is the number of scattered genes in the noise cluster; *C *= {*C*_1_, . . ., *C_k_*, *C_s_*} is the resulting clustering assignment. Minimization of Equation 9 produces a clustering solution. Intuitively, a smaller *λ *will produce tighter clusters, but more genes will be assigned to the noise set.

The prior knowledge comes in the form of *L *known pathways in which gene *i *participates. If each pathway *l *(1, . . ., *L*) contains *N_l _*genes then *x_nl _*is the expression profile of gene *n *in pathway *l*. The value of the weight function *w(x_i_; L) *is directly proportional to the distance between the expression vector of gene *i *(i.e. *x_i_*) and one of the *l *pathways. Thus, the value of *w(xi; L) *is small for genes whose expression vector *xi *closely follows at least one of pathways in the set *L*. How well a gene follows pathways in the set *L *is defined by formula

(10) $$\underset{l}{\min}\frac{1}{N_{l}}\overset{N_{l}}{\underset{n=1}{\sum }}||x_{i}-x_{nl}||$$

Shen and co-authors observed that the parameter *w(xi; L) *in PW-Kmeans algorithm is gene-specific and remains the same no matter which cluster the gene is assigned to [20]. Therefore, while weighting does help identifying the scattered genes, it does not enhance the clustering of genes with similar functions. To overcome this limitation, Shen proposed a novel weighted clustering method, Dynamically Weighted Clustering with Noise set (DWCN) that considers the same weight for all genes within one cluster. Instead of the parameter *w(xi; L) *in the original equation (9) Shen uses the smallest p-value of over representation of all possible GO terms for the genes in the cluster. Consequently, the method separates scattered genes and makes use of functional annotation data to enhance the clustering of genes with similar functions. The authors showed that DWCN outperforms both the original K-means and PW-Kmeans methods on simulated data and gave clusters with strong biological explanation.

The third concept also uses grouping of genes according to prior knowledge in purpose of better clustering. It extends model-based clustering by using the assumption that genes with similar GO annotation have the same probability to belong to one cluster. The concept was realized by Pan [21] in stratified model-based clustering method (Figure 6 in Additional file 1). Model-based clustering methods build a gene probability distribution function to belong to all possible clusters and use the similarity among the functions to cluster genes. The initial probability distribution function for each gene to belong to the clusters is

(11) $$f\left(x_{i};\Theta \right)=\pi \overset{C}{\underset{c=1}{\sum }}f_{c}\left(x_{i};\theta_{c}\right)$$

where *x_i _*is the expression vector of gene *i*, *C *is the number of clusters, *f_c _*is a probability distribution function with parameters $\theta_{c}(\theta_{c}=\left\{\mu_{c},\delta_{c}^{2}\right\}$ where *μ_c _*is a gene expression mean and $\delta_{c}^{2}$ is a gene expression variance in probability distribution *c*). The parameter *π *is the prior probability that a gene originates from each distribution (in other words, the prior probability that a gene belongs to each cluster). The parameter Θ is a set of unknown parameters (*π*, *θ_c_*) that will be maximized in the procedure. Originally, *π *is assumed to be the same for all genes. Pan suggested to take an advantage of known grouping of genes and assign to all genes in each group a prior probability of belonging to a cluster. He replaces *π *by a cluster and gene group specific probability *π_h_*. For that all genes are grouped to *H*_1_, . . . *H subscripth *groups. Then, the same prior probability *π_h _*to end up in one cluster *c *is assigned to all genes in a group *h*. The initial probabilistic function for any gene *i *in functional group *h *became

(12) $$f_{h}\left(x_{i};\Theta_{h}\right)=\overset{C}{\underset{c=1}{\sum }}\pi_{h}f_{c}\left(x_{i};\theta_{c}\right)$$

where Θ*h *= {*π_h_*, *θ_c_*}. Pan argued that the probability component of model-based methods fits very well the highly variable nature of biological data and gives a broad range of possibilities to include biological prior knowledge. As an example, Pan tested the probability of genes with the same GO labeling to comprise one cluster.

The discussed implementations of the second and third concepts do not realize the slider and do not allow to change the ratio between influence of prior knowledge and experimental data. Considering incompleteness and shortcomings of secondary databases that are used in the methods, new realizations of the slider are of interest.

We have summarized cluster methods that include prior knowledge in calculation of the similarity score in Table 2 Additional file 2. All the methods described are from transcriptomics studies. We are not aware of any implementation of cluster models in metabolomics that includes prior knowledge. However, clustering of metabolomics data is a helpful and popular approach. No doubt it is worthwhile to implement clustering methods in metabolomics that are driven by prior knowledge. The functional annotation of metabolites is available and could potentially be used for knowledge guided clustering.

### Supervised classification methods

The main goal of supervised methods is to infer a classification function from a labeled training dataset. The classification function should be able to correctly predict labels of new samples. Examples of such algorithms include regression analysis, support vector machine and decision trees. We define three concepts of including prior knowledge that are used to adjust supervised methods. The first concept separates all variables to groups according to prior knowledge and builds a classification model for each group independently. The second concept forces genes or metabolites that are connected in a network to have close coefficients in the classification function. The third concept uses prior knowledge to predefine the topology of a decision tree.

To reduce the multiple testing problem and to improve the sensitivity and specificity of the classification, the first concept uses a group of related variables to classify the samples. A group may represent a pathway or a set of genes with similar GO annotation. The concept does not require the changes to be in the same direction (only up or only down) but it gives a larger score to a group where changes among more variables are found (Figure 7 in Additional file 1).

The idea was suggested by Goeman *et al*. and implemented in the global test [22]. The authors employed the logistic regression model and rewrote it for *J *samples and *I' *genes as follow

(13) $$E\left(Y_{j}\text{|}\;\beta \right)=h^{-1}\left(\alpha +\overset{I^{\prime }}{\underset{i=1}{\sum }}x_{ij}\beta_{i}\right)$$

where *α *is the intercept, *β_i _*the regression coefficient for gene *i*, *h *the logit function, *x_ij _*is the gene *i *expression profile and *j *is the index for the samples (*j *= 1, . . ., *J*). Note, that the model is built for *I' *variables (genes), which belong to the same group (or pathway). For each group of genes, defined by the prior knowledge, a separate model will be build. The authors suggested a "gene influence" plot to uncover the influence of a single gene. As an example, the authors demonstrated the new method using gene expression data for a cell line treated and untreated with heat shock. While the overall expression profile was not notably different between two groups, the global test showed significant differences for groups of genes known to function in heat shock response according to GO database.

A possibility to use the global test for a metabolomics application was shown by Hendrickx *et al*. [23]. The authors successfully tested a selection of pathway metabolites from KEGG on metabolites profiles of *E. coli *and *S. cerevisiae*. Specifically, they showed that glycolysis pathway and the TCA cycle pathway are significantly different when aerobic conditions are compared to anaerobic conditions for *S. cerevisiae*. The authors concluded that the results of the global test correspond with the physiology of studied organisms and therefore can be used in metabolomics.

The idea of the global test was further developed by several authors including the highly cited method of Chuang *et al*. [24]. They interpret groups of genes as subnetworks and assume that proteins that are close in protein-protein interaction networks have a similar gene expression vector. While the idea to test a group of genes simultaneously instead of multiple testing for each single gene remains the same in Chuang's work, we would like to put attention on how the groups were defined. The authors score subnetworks of protein-protein interaction network in gene expression data of metastatic and non-metastatic breast tumors. To find subnetworks a greedy search algorithm is used. A score function is calculated for a set of genes that is combined based on topology of the network. In each iteration step a next closest gene is added and a new score is checked for increasing. The score function *O(l) *for a particular subnetwork *l *is calculated by the formula

(14) $$O\left(l\right)=\underset{x\in l}{\sum }\underset{y\in z}{\sum }p\left(x,y\right)log\frac{p\left(x,y\right)}{p\left(x\right)p\left(y\right)}$$

where *p(x, y) *is the joint probability density function of subnetwork *l *and a set of output labels *z *(metastatic or non-metastatic); *p(x) *and *p(y) *are marginal density functions. The score function *O(l) *represents the mutual information between gene expression vector *x_i _*from subnetwork *l *over samples and a corresponding vector of sample labels *z*. All significantly different subnetworks represented as gene groups were used to train the logistic regression model. The authors showed that subnetwork markers are more reproducible and achieve higher accuracy in the classification than individual marker genes selected without network information.

The first concept incorporates prior information as a vector with binary labels that show whether or not a gene belongs to a group. This concept does not allow implementation of the slide ruler.

The second concept is based on the idea that genes in the same regulatory network will have similar expression profiles. Following this idea Rapoport *et al*. [25] suggested to consider a gene expression profile from one microarray experiment as a function and to apply the Fourier transform to it. Genes were arranged according to the topology of metabolic networks from the KEGG database. The Fourier transform was used to decompose the expression function into "low-frequency" and "high-frequency" components. The authors argued that the "high-frequency" components contained expression profiles of unimportant genes and measurement errors while the "low-frequency" components reflected properties of the system. The low-frequency part of the decomposed data was successfully used for PCA analysis and to train support vector machine classifiers to distinguish between irradiated and non-irradiated samples of *S. cerevisiae *strains.

Another attempt to implement the second concept was done by Li *et al*. in a network-constrained regularization procedure for a linear regression model [26]. The method requires a smoothness of the regression coefficients across the network. The smoothness means that two variables that are connected in the network must have close weights in the classification function (Figure 8 in Additional file 1). The regularization is based on the normalized Laplacian of the network and similar to *L_1 _*and *L_2 _*penalties on the regression coefficients called the LASSO or elastic net [27] (Figure 9 in Additional file 1 shows an example of Laplacian matrix). For nonnegative penalty coefficients *λ*_1 _and *λ*_2 _the network constrained regularization criterion is defined as follows:

(15) $$\underset{\beta }{\min}\left[{\left(y-X\beta \right)}^{T}\left(y-X\beta \right)+\lambda_{1}\text{|}\beta \text{|}+\lambda_{2}\beta^{T}Lp\beta \right]$$

In the minimization procedure of *β^T^***Lp***β *only coefficients of connected genes are important and coefficients of not connected genes are neglected by 0 in the Laplacian. Moreover, because the sum of each row of the Laplacian is zero, absolute values in *β *which are close in the network are forced to be similar. This is how the network-constrained coefficient *β^T^***Lp***β *induces a smooth solution of *β *on the known network.

The third concept employs a pathway topology to build an easy interpretable decision tree (Figure 10 in Additional file 1). Each inner node corresponds to a gene; each edge corresponds to either up regulation or down regulation of the gene. Finally, each leaf corresponds to a class in the classification problem. By the idea, each path from the root to the leafs can be analyzed for biological interpretation of the system. Consequently, it is possible to analyze the final decision tree and identify up and down regulated genes in each of discriminated classes. The concept was implemented by Dutkowski and Ideker in the method Network-guided forest [28]. It is important to mention that the method is not forced to use all information about the network; only the important for studied experiment and classification problem part will be used. It is of the interest to implement the method in metabolomics, because the network guided forest method uses the network topology but does not assume a similar concentration of neighboring metabolites. The concentration freehold can be used as the decision value.

We summarize supervised methods that include prior knowledge to guide the analysis in Table 3 Additional file 2.

### Covariance matrices

This section provides a separate discussion of the covariance matrix because it plays a central role in many multivariate data analysis methods, as discussed in the previous sections.

Estimation of the covariance matrix from omics data with a low number of samples and a high number of variables is notoriously difficult. A solution is to regularize the estimation by a structured so-called target matrix. Schafer and Strimmer first gave an overview of the most widely used target matrix **Tt **for analysis of high dimensional genomics data that, however, did not incorporate prior knowledge [29]. The authors suggested that the covariance matrix **T **can be estimated as

(16) T=λTt+(1-λ)Tu

where **Tu **is unstructured covariance matrix estimated from data; **Tt **is the structured covariance target matrix. Later, several authors suggested to use prior knowledge to define **Tt **to allow the regularization of all variables in one biological group together rather then individual regularization for each variable [30,31]. We note that if **Tu **represents experimental data and **Tt **represents prior knowledge then *λ *provides an implementation of the slide ruler. The main concept of covariance matrix optimization by prior knowledge is to push the structure of the matrix towards known biology. For example, the confidence that a covariation between two genes in experimental data is not due to the high dimensionality of the data is higher when there is also evidence of a connection between these genes from the prior knowledge (Figure 11 in Additional file 1). We discuss two methods of defining mymatrixTt by prior knowledge below. Guillemont *et al*. presented a method called graph constrained discriminant analysis (gCDA) that regularized estimation of the gene covariances by the Laplacian matrix **Lp **of a known gene regulation network [31]. The authors defined the target matrix **Tt **as

(17) $$Tt={\left(Lp+U\right)}^{-1}$$

where **U **represents the unit (identity) matrix that stabilizes the covariance matrix **Tt**. We give an example of matrix **Tt **in Figure 12 in Additional file 1. The authors compared performance of the method with gene regulation networks inferred from microarray data (other than the analyzed) and with gene regulation networks obtained from KEGG database. Interestingly, gene regulation networks inferred from microarray data always outperformed gene regulation networks from KEGG.

Tai and Pan also used gene regulation networks to construct a matrix **Tt **with block-diagonal structure [30]. All genes were combined in groups (according to pathways in which genes participate) and represented by matrices on the diagonal of **Tt**. The diagonal values are obtained from the covariance matrix **Tu**. The off-diagonals of genes that are not related are set to 0 while the off-diagonals of related genes are calculated by the formula

(18) $$tt_{ii^{\prime }}=t_{h}\sqrt{tu_{i^{\prime }i^{\prime }}tu_{ii}}$$

where *t_h _*is the covariance mean of all genes in the group *h*; *t_i'i' _*and *t_ii _*are diagonal values obtained from the covariance matrix **Tu**. The block-diagonal covariance matrix constructed this way mathematically represents the idea that genes from the same functional group will have more close covariances than genes from different functional groups. The final covariance matrix **T **was used in classification of simulated and real tumor data by linear discriminant analysis. The classification function based on the new covariance matrix showed a better performance compared to classification functions that were based on covariance matrices regularized by mathematical criteria along. Moreover, the interpretation of the result was improved because the classification function was guided by groups of genes with biological meaningful connection.

The final covariance matrix defined by Tai and Pan was further studied by Jelizarow *et al*. [32]. Specifically, they showed that an arbitrary solution to solve prior knowledge ambiguity affected the classification result. The prior knowledge ambiguity included genes that were in no functional group or genes that were in more then one functional group. The authors compared performance of ten structured matrices **Tt **that solved the ambiguity in ten different ways.

## Discussions and conclusions

In this work, we reviewed data analysis methods that incorporate prior biological knowledge in the definition of the model and the estimation of its parameters. Most of the reviewed methods are developed in the field of transcriptomic and only few are available for metabolomics data. It might reflect the problem of metabolite identification in metabolomics data. It remains hard to assign metabolite names to peaks what leaves us with only a limited number of variables which are known and those for which prior knowledge can be incorporated.

Authors of the methods claim that prior knowledge forces and guides the analysis towards the underlying biology and give more reproducible and reliable result. However, to promote a more wide-spread use of these approaches, much more validation of the results is required. Another factor that limits the further use and development of these methods is the lack of easy accessible implementations of the methods. Most of the algorithms are not available as commercial or open-source software (e.g., as an R package), nor are they available as a web-application or web-service. Since these algorithms are generally complex, it will not be easy for a biologist without mathematical and programming skills to implement any of these methods and use it to analyze the data.

One way forward is to define a common and accepted framework to test methods using prior knowledge. Currently, authors use their own set of data and validation procedures, which makes it very hard to compare the performance of such methods. Such a validation framework is important since recent evidence shows that prior knowledge does not always help to improve the result. For example, the probability model based clustering approach of Pan did not show an improvement when including prior knowledge on a set of 300 gene expression microarrays [21]. However, the method seems to give an improvement when applied to a smaller dataset. The authors suggest that in the large dataset there was enough information in the data itself. Staiger *et al*. showed that a simple aggregation of the expression levels of several genes did not outperform a single gene set to train prognostic classifiers in breast cancer [33]. Four methods were compared, including the method of Chuang *et al*. [24], which is discussed in our paper. The authors specifically evaluated a framework to compare performance of four cluster methods that used prior knowledge. First, protein-protein interaction networks and gene regulatory networks were used as prior knowledge to group genes with each of the four methods. Subsequently, the groups were used as features to train three classification methods (nearest mean classifier, logistic regression, 3 NN classifier). While authors of the four methods claimed to increase the stability of features chosen with prior knowledge and/or to increase classification accuracy, Staiger *et al*. showed that they did not perform better than single-gene based methods. To our knowledge, this is the first attempt to develop such a framework and in our opinion, the development of frameworks for correct comparison of different approaches desperately needs more attention.

In general, we can conclude that more research is needed to understand if and how to optimally apply prior knowledge in data analysis methods. A critical assumption is that the prior knowledge is correct and valid for the data being analyzed. If this assumption does not hold, prior knowledge might produce erroneous results. Moreover, it is necessary to study a role of prior knowledge in the analysis of pathological states when main metabolic and regulatory pathways undergo essential changes and no longer are in agreement with mainstream prior knowledge. For example, changes in metabolic pathways [34], gene regulatory pathways [35], and even massive genomic rearrangements [36] are well known for cancer cells. The question is, does knowledge about normal states of a system is appropriate or helpful for exploration of pathological states of the system?

We reviewed more than twenty methods that represent the current state of high-throughput data analysis by incorporating prior knowledge in transcriptomics and metabolomics. We highlighted features and differences of the methods and the type of prior knowledge that was used. We showed that there is a need for a proper framework which would allow a fair comparison of different methods and would help further understanding of how prior knowledge influences results.

## Authors' contributions

PR was responsible for the overall planning and coordination of the review as well as writing the paper; AHCK and JAW equally contributed to the elaboration of the paper. All authors read and approved the final manuscript.

## Competing interests

The authors declare that they have no competing interests.

## Funding

This study was funded by The Netherlands Bioinformatics Centre http://www.nbic.nl. JAW acknowledges funding from STATegra the Seventh Framework Programme [FP7/2007-2013] under grant agreement №306000.

Programme [FP7/2007-2013] under grant agreement №306000 and COST-BMBS, Action BM1006 "Next Generation Sequencing Data Analysis Network", SeqAhead.

## Supplementary Material

Additional file 1 **1. The file contains additional Figures 2 to 12**. 2. Consensus PCA 3. The Network Component Analysis 4. Extending hierarchical clustering by prior knowledge 5. Extending the K-means clustering by pathway information 6. Extending model-based clustering by prior knowledge 7. The global test 8. Extending linear regression model by prior knowledge 9. Example of Laplacian matrix 10. Extending decision tree method by prior knowledge 11. Example of the covariance matrix 12. Example of the structured covariance target matrix. Click here for file

Additional file 2 **The file contains tables 1 to 3 and List of symbols**. 1. Table 1 - Overview of methods that are based on PCA and include prior knowledge. 2. Table 2 - Overview of clustering methods that include prior knowledge. 3. Table 3 - Overview of supervised methods that include prior knowledge to guide the analysis. 4. Table 4 - List of symbols. Click here for file

## Declarations

The publication costs for this article were funded by STATegra the Seventh Framework.

This article has been published as part of BMC Systems Biology Volume 8 Supplement 2, 2014: Selected articles from the High-Throughput Omics and Data Integration Workshop. The full contents of the supplement are available online at http://www.biomedcentral.com/bmcsystbiol/supplements/8/S2.
