# Supplementary material for: Use of prior knowledge for the analysis of high-throughput transcriptomics and metabolomics data
Source: BMC Syst Biol. 2014 Mar 13;8(Suppl 2):S2. doi: 10.1186/1752-0509-8-S2-S2 (PMC4101693; doi:10.1186/1752-0509-8-S2-S2)
Supplement: Additional file 1 — 1. The file contains additional Figures 2 to 12. 2. Consensus PCA 3. The Network Component Analysis 4. Extending hierarchical clustering by prior knowledge 5. Extending the K-means clustering by pathway information 6. Extending model-based clustering by prior knowledge 7. The global test 8. Extending linear regression model by prior knowledge 9. Example of Laplacian matrix 10. Extending decision tree method by prior knowledge 11. Example of the covariance matrix 12. Example of the structured covariance target matrix. [file 1752-0509-8-S2-S2-S1.pdf]

Supplementary Material. Additional figures.

This file belongs to the paper P.Reshetova *et al.* **Use of prior knowledge for the analysis of high-throughput transcriptomics and metabolomics data** and contains additional figures 2 to 12.

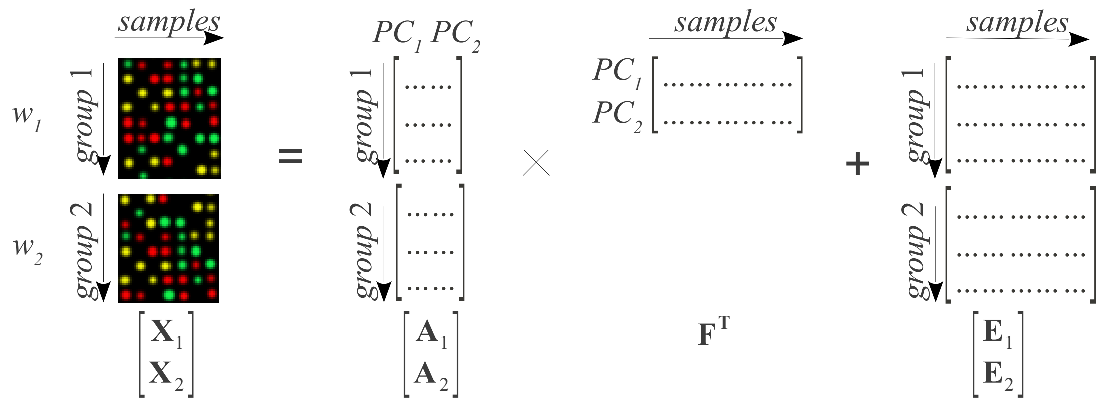

Figure 2: Consensus PCA. The matrix $\mathbf{X}$ with I metabolites and J samples is divided in two matrices $\mathbf{X}_1$ and $\mathbf{X}_2$. Result of the decomposition is the matrix $\mathbf{A}$ that has two parts $\mathbf{A}_1$ and $\mathbf{A}_2$. Each part of the matrix $\mathbf{A}$ describes variations only in the respective part of the matrix $\mathbf{X}$.

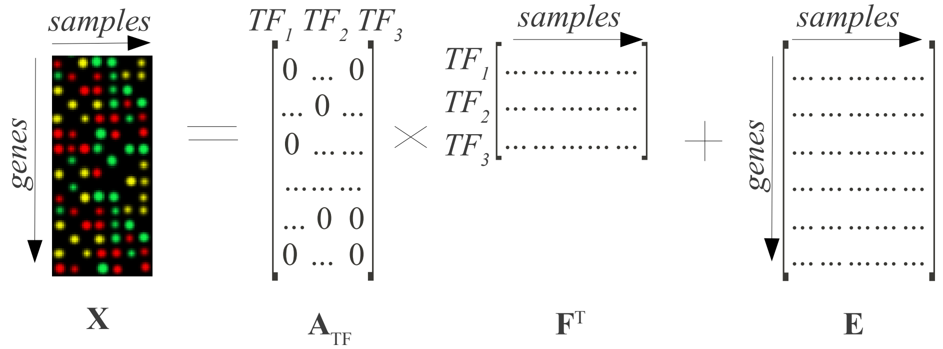

Figure 3: The Network Component Analysis. The matrix $\mathbf{A}_{TF}$ is predefined to represent transcription factors by columns. A column in $\mathbf{A}_{TF}$ has zeros for genes which are not regulated by a specific transcription factor. Values of the regulated genes are estimated. The values in the matrix $\mathbf{F}^T$ are considered as the transcription factors' activity in each sample.

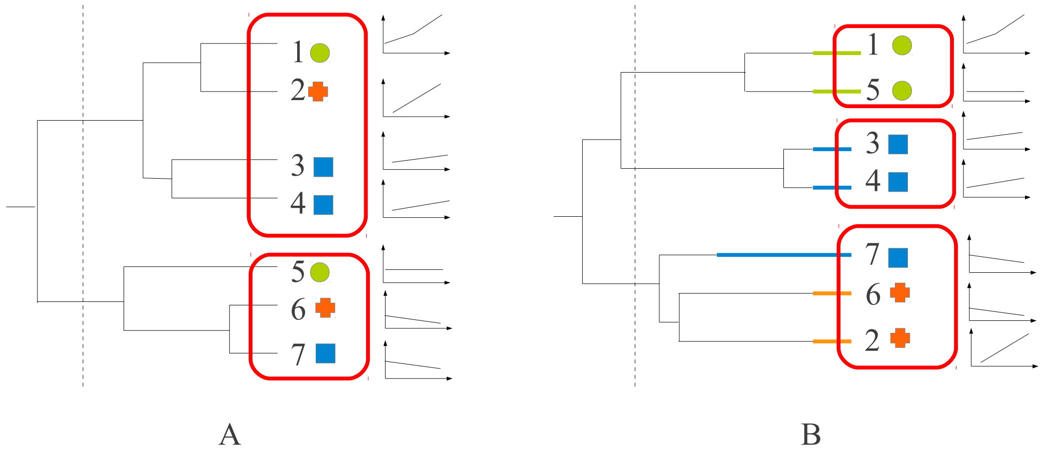

Figure 4: Extending hierarchical clustering by prior knowledge. The difference between hierarchical clustering without (A) and with (B) prior knowledge. Colored figures (circle, cross, and square) indicate GO labels. Red squares indicate clusters. The result of clustering with prior knowledge (B) combines genes with similar expression profiles and similar GO labeling in one cluster.

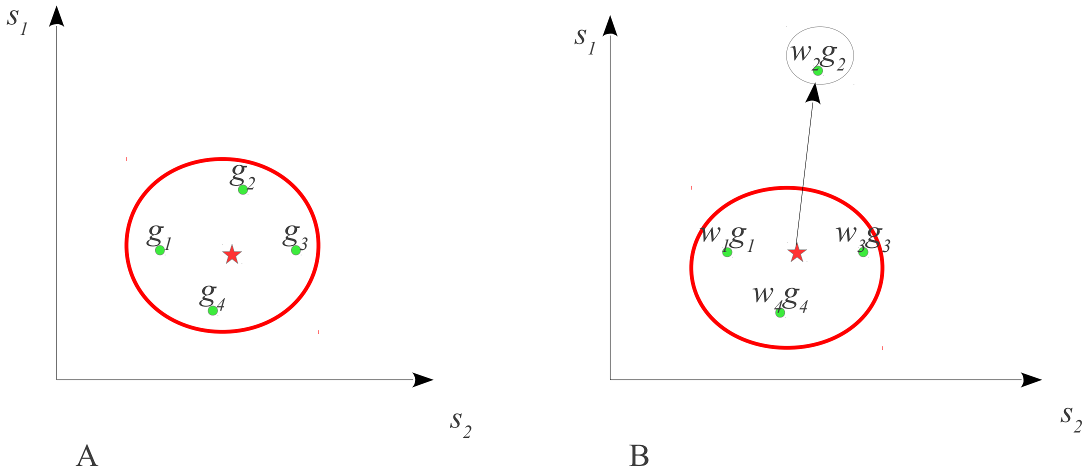

Figure 5: Extending the K-means clustering by pathway information. (A) shows the k-means clustering of four genes based on the gene expression profile similarities s in two samples s_1 and s_2. (B) shows k-means clustering that is extended by the prior knowledge. Distances between gene i and the cluster mean are multiplied by the gene specific weight w_i. w_1, w_3 and w_4 are close to 1 and do not change the distance to the cluster mean greatly. w_2 is big enough to push g_2 from the red cluster to the cluster of scattered genes c_S (c_S is showed by gray color).

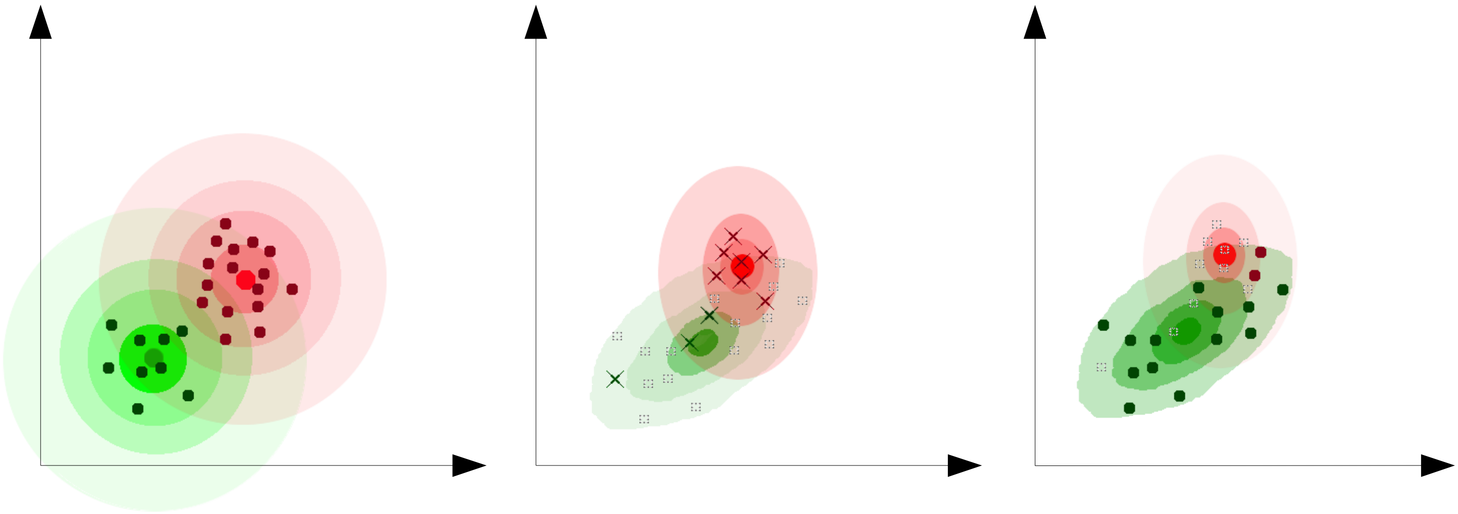

A. B. C.

Figure 6: Extending model-based clustering by prior knowledge. Three mixture models based on two components (red and green circles). Intensity of the colors shows a combination of the weight of each component in a model and posterior probability for a particular variable. Model (A) treats all variables equally and simultaneously. By color of each dot the assigned cluster is shown. For models (B) and (C) variables are split into two groups G_1 (crosses) and G_2(circles). Model (B) is built for group of variables G_1 and the red component has a larger weight in the model. Model (C) is build for group G_2 and the green component has a larger weight in the model. Note that components parameters (the mean and dispersion) in both models stay the same

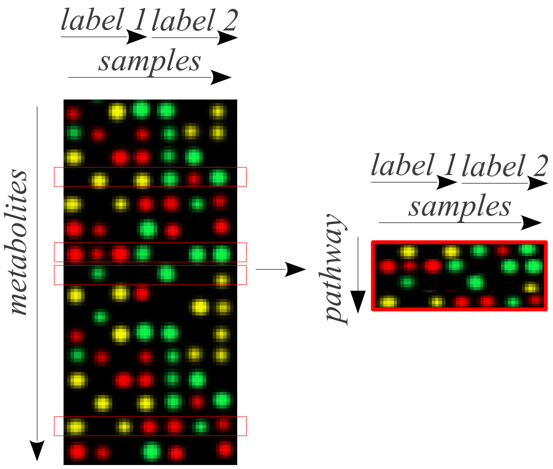

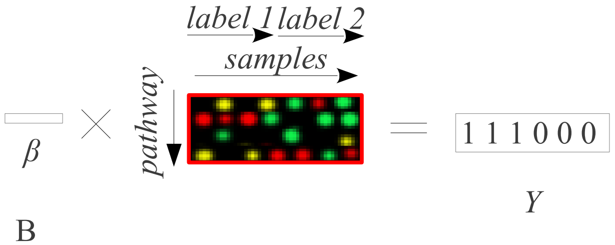

Figure 7: The global test. (A) The first concept selects a group of metabolites according to prior knowledge. (B) Next, a regression model is built for the metabolites in the group. β is a vector of the regression coefficients for each metabolite in the group and it is checked for an association with outcome labels.

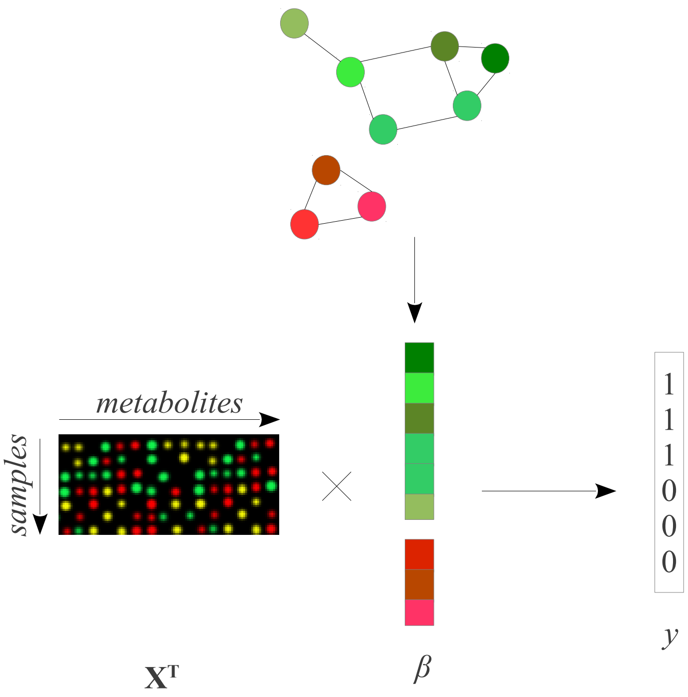

Figure 8: Extending linear regression model by prior knowledge. β is a vector of regression coefficients and it is optimized to be smooth along networks N_1 and N_2.

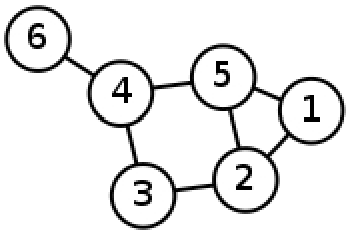

$$\begin{pmatrix} 2 & -1 & 0 & 0 & -1 & 0 \\ -1 & 3 & -1 & 0 & -1 & 0 \\ 0 & -1 & 2 & -1 & 0 & 0 \\ 0 & 0 & -1 & 3 & -1 & -1 \\ -1 & -1 & 0 & -1 & 3 & 0 \\ 0 & 0 & 0 & -1 & 0 & 1 \end{pmatrix}$$

A B

Figure 9: Example of Laplacian matrix. (A) shows a pathway, B shows the corresponding Laplacian matrix. On the diagonal of this matrix the number of links from a specific node can be found. Existence of a link between two nodes coded as -1 in the corresponding place in the Laplacian matrix $\mathbf{Lp}$. This makes the sum of each row and each column equal to 0.

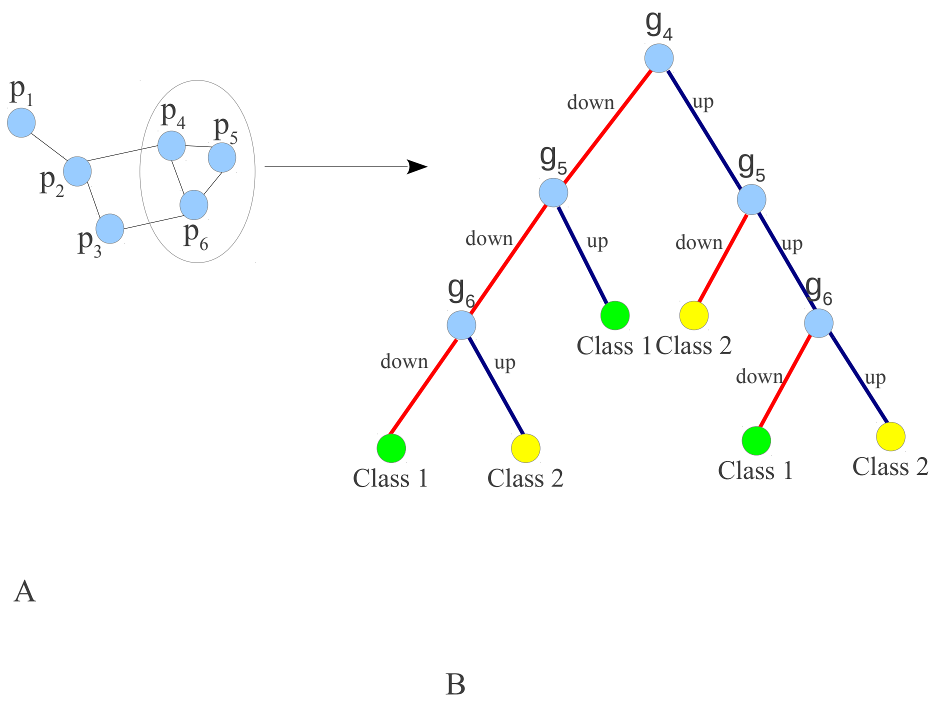

Figure 10: Extending decision tree method by prior knowledge. (A) is a priori known protein interaction network. The method searches for connected network modules and based on them builds decision trees. The gray circle shows an example of such module. (B) is a decision tree that is built based on the network module. For that each protein is assigned to the correspondent gene. Each inner node corresponds to a gene; each edge corresponds to either up regulation or down regulation of the gene. Each leaf corresponds to a class in the classification problem.

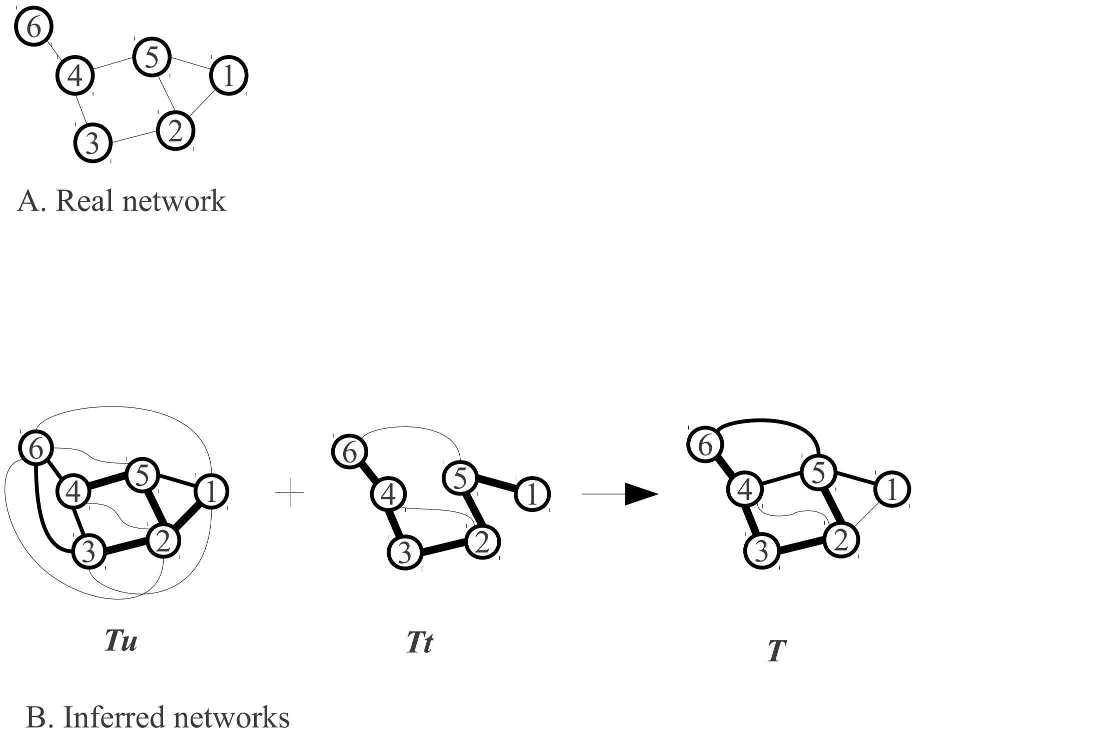

Figure 11: Example of the covariance matrix. Graphs are inferred from different covariance matrices. (A) is a real pathway. (B) shows networks that are inferred from unstructured covariance matrix **Tu** (based on experimental data), structured target covariance matrix **Tt** (based on prior knowledge), and final covariance matrix **T** (based on combination of gene expression values and prior knowledge). Prior knowledge removes false positive links and emphasize known *a priori* links.

$$\left(\underbrace{\begin{pmatrix} 2 & -1 & 0 & 0 & -1 & 0 \\ -1 & 3 & -1 & 0 & -1 & 0 \\ 0 & -1 & 2 & -1 & 0 & 0 \\ 0 & 0 & -1 & 3 & -1 & -1 \\ -1 & -1 & 0 & -1 & 3 & 0 \\ 0 & 0 & 0 & -1 & 0 & 1 \end{pmatrix}}_{\text{Lp}} + \underbrace{\begin{pmatrix} 1 & 0 & 0 & 0 & 0 & 0 \\ 0 & 1 & 0 & 0 & 0 & 0 \\ 0 & 0 & 1 & 0 & 0 & 0 \\ 0 & 0 & 0 & 1 & 0 & 0 \\ 0 & 0 & 0 & 0 & 1 & 0 \\ 0 & 0 & 0 & 0 & 0 & 1 \end{pmatrix}}_{\text{U}}\right)^{-1} = \underbrace{\begin{pmatrix} 0.45 & 0.18 & 0.08 & 0.07 & 0.17 & 0.03 \\ 0.18 & 0.37 & 0.15 & 0.09 & 0.16 & 0.05 \\ 0.08 & 0.15 & 0.43 & 0.15 & 0.10 & 0.08 \\ 0.07 & 0.09 & 0.15 & 0.37 & 0.13 & 0.18 \\ 0.17 & 0.16 & 0.10 & 0.13 & 0.37 & 0.07 \\ 0.04 & 0.04 & 0.08 & 0.18 & 0.07 & 0.59 \end{pmatrix}}_{\text{Tt}}$$

Figure 12: Example of the structured target covariance matrix **Tt** in graph constracted discriminant analysis of Guillemont *et al.*. This example is constructed for the network shown in Figure 9. When two nodes are connected in the graph, the covariance (off diagonals) is expected to be higher than the not connected nodes. For the variances in the covariance matrix (diagonal elements) we see that a node connected to many other nodes (e.g. a hub) is expected to have a lower variance than the nodes with few connections. It is a mathematical representation of the biological idea that hub nodes are tightly regulated and thus expected not to vary much in a particular situation.
