# Supplementary material for: Use of prior knowledge for the analysis of high-throughput transcriptomics and metabolomics data
Source: BMC Syst Biol. 2014 Mar 13;8(Suppl 2):S2. doi: 10.1186/1752-0509-8-S2-S2 (PMC4101693; doi:10.1186/1752-0509-8-S2-S2)
Supplement: Additional file 2 — The file contains tables 1 to 3 and List of symbols. 1. Table 1 - Overview of methods that are based on PCA and include prior knowledge. 2. Table 2 - Overview of clustering methods that include prior knowledge. 3. Table 3 - Overview of supervised methods that include prior knowledge to guide the analysis. 4. Table 4 - List of symbols. [file 1752-0509-8-S2-S2-S2.pdf]

Supplementary Material. Tables.

This file belongs to the paper P.Reshetova *et al.* **Use of prior knowledge for the analysis of high-throughput transcriptomics and metabolomics data** and contains tables 1 to 4.

Tables

Table 1 - Overview of methods that are based on PCA and include prior knowledge.

Method	Applied on / Prior knowledge	Principle
Consensus PCA [1]	Metabolom of *P.putida S12* and *E. coli* / set of important metabolites	**A** is divided on two parts, one of which explains variations in a selection of important metabolites and the other explains variations in the rest of metabolites.
NCA [2]	DNA microarrays of *S. cerevisiae* / transcription factors activities	**A** represents a transcription factor by each column and has zeros representing that the specific gene is not regulated by that specific transcription factor. Only elements in **A** that are not restricted to be 0 will be estimated to minimize the sum of the squared residuals in **E**
GCA [3]	DNA microarrays of *S. cerevisiae* / transcription factors targets	**A** represents a transcription factor by each column similar to NCA but the zeros are allowed to be small values. There is also a penalty where $\mathbf{A}^{true}$ is the structure as applied in NCA and the method allow **A** be different from $\mathbf{A}^{true}$ according to the penalty

Table 2 - Overview of clustering methods that include prior knowledge.

Method	Applied on / Prior knowledge	Principle
Cheng et al [18].	The top 80 ranked genes in DNA microarrays according to F-scores in Leukocyte differentiation time-series experiment on mouse. / Similarity between two GO classes according to the topology of GO tree.	The similarity score between two genes is the sum of the GO annotation similarity and gene expression profile similarity.
R. Kustra, A. Zagdański [19].	3224 yeast genes from 424 microarray experiments / Information Content between two GO terms	The similarity score between two genes is a sum of the GO annotation similarity and the gene expression profile similarity. But the contribution of each part is specifically defined by the λ parameter
Penalized and Weighted K-means clustering (PK-means clustering) [4].	Mass spectrometry data of 2856 peptides of 22 amino acids long. DNA microarrays from *S. cerevisiae* cell-cycle dataset (from Spellman) / Gene functional annotations (GO)	Combine genes with a similar annotation and an expression profile in one cluster and create a cluster of scattered genes
Dynamically Weighted Clustering with Noise [5].	DNA microarrays from *S. cerevisiae* cell-cycle dataset and 112 segregants in a cross between two parental strains BY and RM / Gene functional annotations (GO)	Combine genes with similar annotation and expression profile in one cluster and and create a cluster of scattered genes. As opposed to PK-means method, each cluster has its own set of terms in the annotation.
Probability model-based clustering [6].	300 microarray experiments with gene deletions and drug treatments for *S. cerevisiae.* / GO functional annotations	Assign same prior probability of belonging to one cluster to all genes which are labeled by the same GO term.
Co-clustering of genes and vertices in the network [7].	DNA microarrays of seven time points for *S. cerevisiae.* After mapping to KEGG database 1571 genes and proteins were clustered / Metabolic pathways	Assign a similarity value to pairs of genes based on their distance in a network and expression the profile similarity
Hierarchical tree snipping [8].	DNA microarrays for *S. cerevisiae* cell-cycle experiment / GO annotations	Put genes which are close in the cluster tree and with similar GO annotation in one cluster by allowing cut clusters in different tree levels.

Table 3 - Overview of supervised methods that include prior knowledge to guide the analysis.

Method	Applied on / Prior knowledge	Principle
Global test [9]	microarray data of 3571 genes from 27 patients with Acute Lymphoic Leukemia and 11 patients with Acute Myeloid Leukemia. In-house 20160 oligonucleotides array for a cell line treated/untreated with a heat shock. / Groups of variables	Test if the mean of all variables in a group is related to different experimental conditions.
Global test in metabolomics [10]	metabolome of E. coli measured by LC-MS, GC-MS; LC–MS data of *S. cerevisiae* / Metabolic pathways	Test if the mean of all variables in a group is related to different experimental conditions.
Network-based classification [11]	Microarrays of metastatic and non-metastatic breast tumor tissues. / Protein-protein interaction network.	Define distinguishable for an outcome subnetworks, by testing the mean of expression of all genes in the subnetworks. Use the distinguishable subnetworks to train a classifier.
Network based decomposition of gene expression data [12]	Microarrays of irradiated and non-irradiated *S. cerevisiae* strains / metabolic pathways	Remove the high frequent component from gene expression profiles according to the topology of gene regulation pathways.
Li et al [13]	DNA microarrays of glioblastoma samples / Gene regulation networks	Define a network-constrained penalty function for linear regression model to make the coefficients smooth on the network Network-guided forest
Network-guided forest [14]	DNA microarrays of of germ samples, breast and brain cancer samples / Protein-protein interaction networks.	Build a classifier as classification tree based on a protein-protein interaction network topology.

Table 4 - List of symbols.

Symbol	Meaning
$\mathbf{X}$ (I x J)	Data matrix
I, i = 1,...,I	Number of genes or metabolites
J, j = 1,...,J	Number of samples
x_i (1 x J)	Gene expression vector
$\mathbf{A}$ (IxR)	Score matrix in data decomposition methods
R , r = 1,...,R	Number of components in decomposition methods
$\mathbf{F}$ (J x R)	Loading matrix in data decomposition methods
$\mathbf{E}$ (I x J)	Residuales matrix in data decomposition methods
w	Weights in consensus PCA
$\mathbf{W}$ (I x R)	Indicator matrix in GCA
$\mathbf{A}^{true}$ (I x R)	Matrix predefined by a priory known transcription factors regulation for each gene.
$\mathbf{S}$ (I x I)	Matrix of similarity scores between genes based on experimental data
$\mathbf{G}$ (I x I)	Matrix of similarity scores between genes based on prior knowledge
$\mathbf{D}$ (I x I)	Matrix of similarity scores between genes based on combination of experimental data and prior knowledge
$\mathbf{C}$ (I x K)	Cluster matrix
K, k = 1,...,K	Number of clusters
C_S	Cluster that contains scattered variables
$\|S\|$	Number of scattered variables in cluster C_S
L (1 x L), l = 1, ..., L	Pathways
N_l, n = 1, ..., N_l	Number of genes in pathway l
x_{nl}	expression profile vector of gene n in pathway l
H, h = 1, ..., H	gene groups defined by prior knowledge
**Lp**	Laplacian matrix
**Tu**	Covariance matrix based on experimental data
**Tt**	Covariance matrix based on prior knowledge
**T**	Covariance matrix based on experimental data and prior knowledge
t_h	mean of covariances between genes in group h
**U**	Unit (identity) matrix

References

1. van den Berg RA, Rubingh CM, Westerhuis JA, van der Werf MJ, Smilde AK: **Metabolomics data exploration guided by prior knowledge**. *Analytica Chimica Acta* 2009, **651**(2):173–181.
2. Liao JC, Boscolo R, Yang YL, Tran LM, Sabatti C, Roychowdhury VP: **Network component analysis: reconstruction of regulatory signals in biological systems**. *Proceedings of the National Academy of Sciences of the United States of America* 2003, **100**(26):15522–15527.
3. Westerhuis JA, Derks EPPA, Hoefsloot HCJ, Smilde AK: **Grey component analysis**. *Journal of Chemometrics* 2007, **21**(10-11):474–485.
4. Tseng GC: **Penalized and weighted K-means for clustering with scattered objects and prior information in high-throughput biological data**. *Bioinformatics* 2007, **23**(17):2247–2255.
5. Shen Y, Sun W, Li KC: **Dynamically weighted clustering with noise set**. *Bioinformatics* 2009, **26**(3):341–347.

6. Pan W: **Incorporating gene functions as priors in model-based clustering of microarray gene expression data**. *Bioinformatics (Oxford, England)* 2006, **22**(7):795–801.
7. Hanisch D, Zien A, Zimmer R, Lengauer T: **Co-clustering of biological networks and gene expression data**. *Bioinformatics (Oxford, England)* 2002, **18 Suppl 1**:S145–154.
8. Dotan-Cohen D, Melkman AA, Kasif S: **Hierarchical tree snipping: clustering guided by prior knowledge**. *Bioinformatics (Oxford, England)* 2007, **23**(24):3335–3342.
9. Goeman JJ, van de Geer SA, de Kort F, van Houwelingen HC: **A global test for groups of genes: testing association with a clinical outcome**. *Bioinformatics* 2003, **20**:93–99.
10. Hendrickx DM, Hoefsloot HC, Hendriks MM, Canelas AB, Smilde AK: **Global test for metabolic pathway differences between conditions**. *Analytica Chimica Acta* 2012, **719**:8–15.
11. Chuang HY, Lee E, Liu YT, Lee D, Ideker T: **Network-based classification of breast cancer metastasis**. *Molecular Systems Biology* 2007, **3**.
12. Rapaport F, Zinovyev A, Dutreix M, Barillot E, Vert JP: **Classification of microarray data using gene networks**. *BMC Bioinformatics* 2007, **8**:35.
13. Li C, Li H: **Network-constrained regularization and variable selection for analysis of genomic data**. *Bioinformatics (Oxford, England)* 2008, **24**(9):1175–1182.
14. Dutkowski J, Ideker T: **Protein networks as logic functions in development and cancer**. *PLoS computational biology* 2011, **7**(9):e1002180.
